# Extracting conflict-free information from multi-labeled trees

**DOI:** 10.1186/1748-7188-8-18

**Published:** 2013-07-09

**Authors:** Akshay Deepak, David Fernández-Baca, Michelle M McMahon

**Affiliations:** 1 Department of Computer Science, Iowa State University, Ames, Iowa, USA; 2 School of Plant Sciences, University of Arizona, Tucson, Arizona, USA

**Keywords:** Phylogenetic trees, Evolutionary trees, Multi-labeled trees, Reduction, Singly-labeled trees

## Abstract

**Background:**

A multi-labeled tree, or MUL-tree, is a phylogenetic tree where two or more leaves share a label, e.g., a species name. A MUL-tree can imply multiple conflicting phylogenetic relationships for the same set of taxa, but can also contain conflict-free information that is of interest and yet is not obvious.

**Results:**

We define the information content of a MUL-tree *T* as the set of all conflict-free quartet topologies implied by *T*, and define the maximal reduced form of *T* as the smallest tree that can be obtained from *T* by pruning leaves and contracting edges while retaining the same information content. We show that any two MUL-trees with the same information content exhibit the same reduced form. This introduces an equivalence relation among MUL-trees with potential applications to comparing MUL-trees. We present an efficient algorithm to reduce a MUL-tree to its maximally reduced form and evaluate its performance on empirical datasets in terms of both quality of the reduced tree and the degree of data reduction achieved.

**Conclusions:**

Our measure of conflict-free information content based on quartets is simple and topologically appealing. In the experiments, the maximally reduced form is often much smaller than the original tree, yet retains most of the taxa. The reduction algorithm is quadratic in the number of leaves and its complexity is unaffected by the multiplicity of leaf labels or the degree of the nodes.

## Background

**Figure 1:**
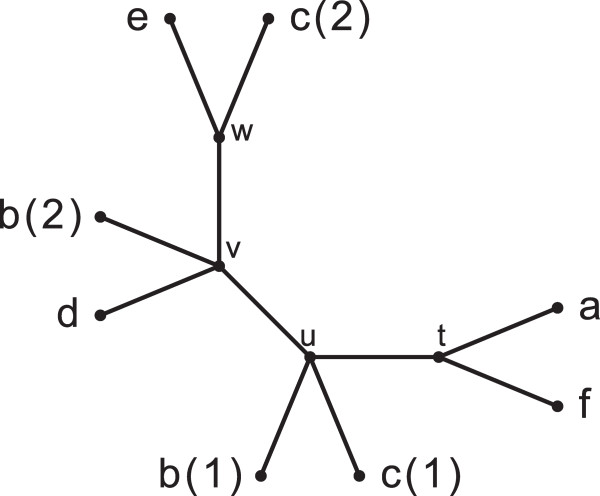
**A MUL-tree.** Numbers in parenthesis next to labels indicate the multiplicity of the respective labels and are not part of the labels themselves.

Multi-labeled trees, also known as MUL-trees, are phylogenetic trees that can have more than one leaf with the same label [1-5] (Figure 1). MUL-trees arise naturally and frequently in data sets containing multiple gene sequences for the same species [6], but they can also arise in biogeographical studies or co-speciation studies where leaves represent individual taxa yet are labeled with their areas [7] or hosts [8].

MUL-trees, unlike singly-labeled trees, can contain conflicting species-level phylogenetic information due to biological processes such as whole genome duplications [9] or incomplete lineage sorting [10], to artifactual processes such as inferential error, or, frequently, an unknown combination of several factors. However, they can also contain substantial amounts of conflict-free information.

Here we provide a way to extract this information; specifically, we have the following results. 

• We introduce a new quartet-based measure of the information content of a MUL-tree, defined as the set of conflict-free quartets that the tree displays (see **MUL-Trees and information content** on page 3).

• We introduce the concept of the maximally-reduced form (MRF) of a MUL-tree *T*, the smallest tree with the same information content as *T* (see **Maximally reduced MUL-Trees** on page 4), and show that any two MUL-trees with the same information content have the same MRF (Theorem 3).

• We present a simple algorithm to construct the MRF of a MUL-tree (see **The reduction algorithm** on page 7). Its running time is quadratic in the number of leaves and does not depend on the multiplicity of the leaf labels or the degrees of the internal nodes.

• We present computational experience with an implementation of our MRF algorithm (see **Results and discussion** on page 8). In our test data, the MRF is often significantly smaller than the original tree, while retaining most of the taxa.

We now give the intuition behind our notion of information content, deferring the formal definitions of this and other concepts to the next section. Quartets (i.e., sets of four species) are a natural starting point, since they are the smallest subsets from which we can draw meaningful topological information. A singly-labeled tree implies exactly one topology on any quartet. More precisely, each edge *e* in a singly-labeled tree implies a bipartition (*A*,*B*) of the leaf set, where each part is the set of leaves on one of the two sides of *e*. From (*A*,*B*), we derive a collection of bipartitions *ab*|*cd* of quartets, such that {*a*,*b*} ⊆ *A* and {*c*,*d*} ⊆ *B*. Clearly, if one edge in a singly-labeled tree implies some bipartition *q* = *ab*|*cd* of {*a*,*b*,*c*,*d*}, then there can be no other edge that implies a bipartition, such as *ac*|*bd*, that is in conflict with *q*. Indeed, the quartet topologies implied by a singly-labeled tree uniquely identify it [11].

The situation for MUL-trees is more complicated, as illustrated in Figure 1. Here, the presence of two copies of labels *b* and *c* — *b*(1) and *b*(2), and, *c*(1) and *c*(2) — leads to two conflicting topologies on the quartet {*b*,*c*,*d*,*e*}. Edge (*u*,*v*) implies the bipartition *bc*|*de*, corresponding to the labels {*b*(1),*c*(1),*d*,*e*}, while edge (*v*,*w*) implies *bd*|*ce* corresponding to the leaves {*b*(2),*c*(2),*d*,*e*}. On the other hand, the quartet topology *af*|*bc*, implied by edge (*t*,*u*), has no conflict with any other topology that the tree exhibits on {*a*,*b*,*c*,*f*}. We show that the set of all such conflict-free quartet topologies is compatible (Theorem 1). That is, for every MUL-tree *T* there exists at least one singly-labeled tree that displays all the conflict-free quartets of *T* — and possibly some other quartets as well. Motivated by this, we only view conflict-free quartet topologies as informative, and define the information content of a MUL-tree as the set of all conflict-free quartet topologies it implies.

We should note that conflicting quartets may well provide valuable information, whether about paralogy, deep coalescence, or mistaken annotations. In some cases, species-level phylogenetic information can be recovered from conflicted quartets through application of, e.g., gene-tree species-tree reconciliation (generally an NP-hard problem [12]). However, this is not feasible when the underlying cause of multiplicity is unknown or when conducting large-scale analyses. Our definition of information content is deliberately designed to make no assumptions about the cause of conflict. It is also conservative with respect to species relationships, i.e., it does not introduce quartets not originally supported by the data. Further, knowing the information content of a MUL-tree allows us to easily identify its conflicting quartets as well.

**Figure 2:**
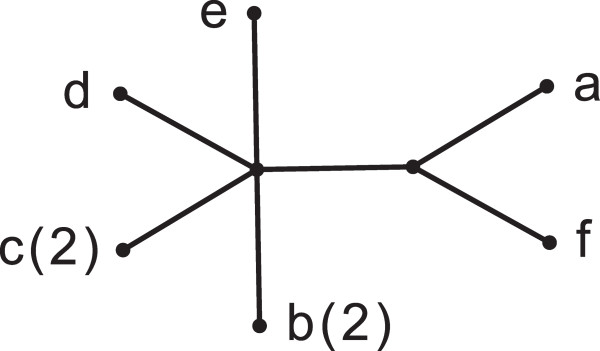
**The MRF for the Mul-tree in Figure**1**.**

A MUL-tree may have leaves that can be pruned and edges that can be contracted without altering the tree’s information content, i.e., without adding or removing conflict-free quartets. For example, in Figure 1, every quartet topology that edge (*v*,*w*) implies is either in conflict with some other topology (e.g., for set {*b*,*c*,*d*,*e*}) or is already implied by some other edge (e.g., *af*|*ce* is also implied by (*t*,*u*)). Thus, (*v*,*w*) can be contracted without altering the information content. In fact, the information content remains unchanged if we also contract (*u*,*v*) and remove the leaves labeled *b*(1) and *c*(1). We define the MRF of a MUL-tree *T* as the tree that results from applying information-preserving edge contraction and leaf pruning operations repeatedly to *T*, until it is no longer possible to do so. The MRF of the tree in Figure 1 is shown in Figure 2. In this case, the MRF is singly-labeled; however, this is not true in general (see **An example** on page 8). If the MRF is itself a MUL-tree, it is not possible to reduce the original to a singly-labeled tree without either adding at least one quartet that did not exist conflict-free in *T* or by losing one or more conflict-free quartets.

Since any two MUL-trees with the same information content have the same MRF, rather than comparing MUL-trees directly, we can instead compare their MRFs. This is appealing mathematically, because it focuses on conflict-free information content, and also computationally, since an MRF can be much smaller than the original MUL-tree. Indeed, on our test data, the MRF was frequently singly-labeled. This reduction in input size is especially significant if the MUL-tree is an input to an algorithm whose running time is exponential in the label multiplicity, such as Ganapathy et al.’s algorithm to compute the contract-and-refine distance between two area cladograms [7] or Huber et al.’s algorithm to determine if a collection of “multi-splits” can be displayed by a MUL-tree [13].

For our experiments, we also implemented a post-processing step, which converts the MRF to a singly-labeled tree, rendering it available for analyses that require singly-labeled trees, including supermatrix [14,15] and supertree methods [16-19]. On the trees in our data set, the combined taxon loss between the MRF computation and the postprocessing was much lower than it would have been had we simply removed all duplicate taxa from the original trees.

Previous work on MUL-trees has concentrated on finding ways to reduce MUL-trees to singly-labeled trees (typically in order to provide inputs to supertree methods) [5], and to develop metrics and algorithms to compare MUL-trees [7,20-22]. In contrast to our approach — which is purely topology-based and is agnostic with respect to the cause of label multiplicity — the assumption underlying much of the literature on MUL-trees is that taxon multiplicity results from gene duplication. Thus, methods to obtain singly-labeled trees from MUL-trees usually work by pruning subtrees at putative duplication nodes. Although the proposed algorithms are polynomial, they are unsatisfactory in various ways. For example, in [5] if the subtrees are neither identical nor compatible, then the subtree with smaller information content is pruned, which seems to discard too much information. Further, the algorithm is only efficient for binary rooted trees. In [20] subtrees are pruned arbitrarily, while in [21] at each putative duplication node a separate analysis is done for each possible pruned subtree. Although the latter approach is better than pruning arbitrarily, in the worst case it can end up analyzing exponentially many subtrees.

## MUL-Trees and information content

A *MUL-tree* is a triple (*T*,*M*,*ψ*), where (i) *T* is an unrooted tree^a^ with leaf set L(T) all of whose internal nodes have degree at least three, (ii) *M* is a set of labels, and (iii) $\psi_{T}:L(T)\to M$ is a surjective map that assigns each leaf of *T* a label from *M*. (Note that if *ψ* is a bijection, *T* is singly labeled; that is, singly-labeled trees are a special case of MUL-trees.) For brevity we often refer to a MUL-tree by its underlying tree *T*. In what follows, unless stated otherwise, by a tree we mean a MUL-tree.

An edge (*u*,*v*) in *T* is *internal* if neither *u* nor *v* belong to L(T), and is *pendant* otherwise. A *pendant node* is an internal node that has a leaf as its neighbor.

Let (*u*,*v*) be an edge in *T* and *T*^′^ be the result of deleting (*u*,*v*) from *T*. Then $T_{u}^{\text{uv}}$ ( $T_{v}^{\text{uv}}$) denotes the subtree of *T*^′^ that contains *u* (*v*). $M_{u}^{\text{uv}}$ ( $M_{v}^{\text{uv}}$) denotes the set of labels in $T_{u}^{\text{uv}}$ ( $T_{v}^{\text{uv}}$) but not in $T_{v}^{\text{uv}}$ ( $T_{u}^{\text{uv}}$). *C*^*uv*^ is the set of labels common to both $T_{u}^{\text{uv}}$ and $T_{v}^{\text{uv}}$. Observe that $M_{u}^{\text{uv}}$, $M_{v}^{\text{uv}}$ and *C*^*uv*^ partition *M*. For example, in Figure 1, $M_{u}^{\text{uv}}=\left\{a,f\right\}$, $M_{v}^{\text{uv}}=\left\{e,d\right\}$, *C*^*uv*^ = {*b*,*c*}.

A (resolved) *quartet* in a MUL-tree *T* is a bipartition *ab*|*cd* of a set of labels {*a*,*b*,*c*,*d*} such that there is an edge (*u*,*v*) in *T* with $\left\{a,b\right\}\in M_{u}^{\text{uv}}$ and $\left\{c,d\right\}\in M_{v}^{\text{uv}}$. We say that (*u*,*v*) *resolves* *ab*|*cd*. For example, in Figure 1, edge (*t*,*u*) resolves *af*|*bc*.

The *information content of an edge* (*u*,*v*) of a MUL-tree *T*, denoted Δ(*u*,*v*), is the set of quartets resolved by (*u*,*v*). An edge (*u*,*v*) in tree *T* is *informative* if |Δ(*u*,*v*)| > 0; (*u*,*v*) is *maximally informative* if there is no other edge (*u*^′^,*v*^′^) in *T* with Δ(*u*,*v*) ⊂ Δ(*u*^′^,*v*^′^). The *information content* of *T*, denoted I(T), is the combined information content of all edges in the tree; that is $I(T)=\underset{(u,v)\in E}{⋃}\Delta (u,v)$, where *E* denotes the set of edges in *T*.

The next result shows that the quartets in I(T) are conflict-free.

### Theorem 1

*For every MUL-tree T, there is a singly labeled tree T*^′^*such that*$I(T)\subseteq I(T^{\prime })$.

### Proof

Repeat the following step until *T* has no multiply-occurring labels. Pick any multiply-occurring label *ℓ* in *T*, select an arbitrary leaf labeled by *ℓ*, and relabel every other leaf labeled by *ℓ*, by a new, unique, label. The resulting tree *T*^′^ is singly labeled, and all labels of *T* are also present in *T*^′^. Consider a quartet *ab*|*cd* in *T*, that is resolved by edge (*u*,*v*). Assume that $\{a,b\}\in M_{u}^{\text{uv}}$ and $\{c,d\}\in M_{v}^{\text{uv}}$. Thus, $T_{u}^{\text{uv}}$ contains all the occurrences of label *a*. Clearly, this also holds for the only occurrence of *a* in *T*^′^. Similar statements can be made about labels *b*, *c*, and *d*. Thus, the quartet *ab*|*cd* is resolved by edge (*u*,*v*) in *T*^′^, and, hence, *T*^′^ displays all quartets of *T*. □

Note that there are examples where the containment indicated by the above result is proper.

**Figure 3:**
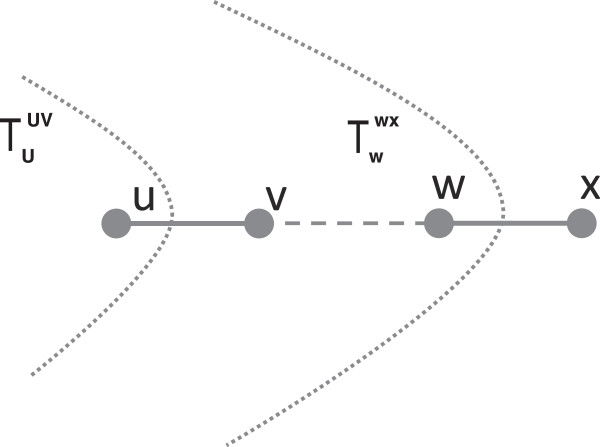
Supportive illustration for the proof of Lemma 1.

To conclude this section, we give some results that are useful for the MUL-tree reduction algorithm (see **The reduction algorithm**, beginning on page 7). In the next lemmas, (*u*,*v*) and (*w*,*x*) denote two edges in tree *T* that lie on the path $P_{u,x}=(u,v,\ldots ,w,x)$ as shown in Figure 3.

### Lemma 1.

*If*$\left|M_{u}^{\text{uv}}|=|M_{w}^{\text{wx}}\right|$*then*$M_{u}^{\text{uv}}=M_{w}^{\text{wx}}$. *Otherwise,*$M_{u}^{\text{uv}}\subset M_{w}^{\text{wx}}$.

### Proof

Refer to Figure 3. Since $T_{u}^{\text{uv}}$ is a subtree of $T_{w}^{\text{wx}}$, $M_{u}^{\text{uv}}\subseteq M_{w}^{\text{wx}}$ by definition of $M_{u}^{\text{uv}}$. Thus, if $\left|M_{u}^{\text{uv}}|=|M_{w}^{\text{wx}}\right|$, we must have $M_{w}^{\text{wx}}=M_{u}^{\text{uv}}$ and, if $\left|M_{u}^{\text{uv}}|\neq |M_{w}^{\text{wx}}\right|$, we must have $M_{u}^{\text{uv}}\subset M_{w}^{\text{wx}}$. □

Together with Lemma 1, the next result allows us to check whether the information content of an edge is a subset of that of another based solely on the cardinalities of the $M_{u}^{\text{uv}}$s.

### Lemma 2

Δ(*u*,*v*) ⊆ Δ(*w*,*x*) *if and only if*$M_{v}^{\text{uv}}=M_{x}^{\text{wx}}$.

### Proof

*(Only if)* Suppose Δ(*u*,*v*) ⊆ Δ(*w*,*x*); therefore, $M_{v}^{\text{uv}}\subseteq M_{x}^{\text{wx}}$. By definition, $M_{v}^{\text{uv}}⊇M_{x}^{\text{wx}}$; hence, $M_{v}^{\text{uv}}=M_{x}^{\text{wx}}$.

*(If)* Suppose $M_{v}^{\text{uv}}=M_{x}^{\text{wx}}$. By definition, $M_{u}^{\text{uv}}\subseteq M_{w}^{\text{wx}}$, which implies that Δ(*u*,*v*) ⊆ Δ(*w*,*x*). □

### Lemma 3

*Suppose* Δ(*u*,*v*) ⊆ Δ(*w*,*x*). *Then, for any edge* (*y*,*z*) *onP*_*u*,*x*_*such thatvis closer toythan toz*, Δ(*u*,*v*) ⊆ Δ(*y*,*z*) ⊆ Δ(*w*,*x*).

### Proof

By Lemma 2, since Δ(*u*,*v*) ⊆ Δ(*w*,*x*), we have $M_{v}^{\text{uv}}=M_{x}^{\text{wx}}$. Now consider an edge (*y*,*z*) on *P*_*u*,*x*_. By definition $M_{v}^{\text{uv}}⊇M_{z}^{\text{yz}}⊇M_{x}^{\text{wx}}$. But $M_{v}^{\text{uv}}=M_{x}^{\text{wx}}$, therefore $M_{v}^{\text{uv}}=M_{z}^{\text{yz}}=M_{x}^{\text{wx}}$. By definition $M_{u}^{\text{uv}}\subseteq M_{y}^{\text{yz}}\subseteq M_{w}^{\text{wx}}$. Hence, by Lemma 2, Δ(*u*,*v*) ⊆ Δ(*y*,*z*) ⊆ Δ(*w*,*x*). □

## Maximally reduced MUL-Trees

Our goal is to provide a way to reduce a MUL-tree *T* as much as possible, while preserving its information content. Our reduction algorithm uses the following operations. Prune (*v*): Delete leaf *v* from *T*. If, as a result, *v*’s neighbor *u* becomes a degree-two node, connect the former two neighbors of *u* by an edge and delete *u*. Contract (*e*): Delete an internal edge *e* and identify its endpoints.

A leaf *v* in *T* is *prunable* if the tree that results from pruning *v* has the same information content as *T*. An internal edge *e* in *T* is *contractible* if the tree that results from contracting *e* has the same information content as *T*. *T* is *maximally reduced* if it has no prunable leaf and no contractible internal edge.

### Theorem 2

Every internal edge in a maximally reduced tree T resolves a quartet that is resolved by no other edge.

### Proof

We rely on two facts. First, every internal node in the tree has degree at least three. Second, every internal edge in the tree resolves a quartet; otherwise, the edge would be contractible and the tree would not be maximally reduced.

**Figure 4:**
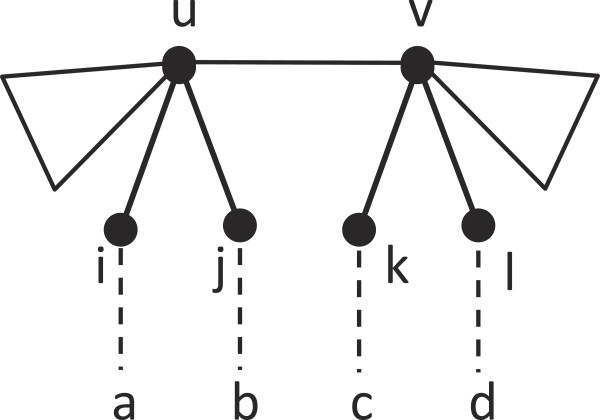
**Supportive illustration for the proof of Theorem 2.** Quartet *ab*|*cd* is resolved only by edge (*u*,*v*). Here, $a\in M_{i}^{\text{ui}}$, $b\in M_{j}^{\text{uj}}$, $c\in M_{k}^{\text{vk}}$ and $d\in M_{l}^{\text{vl}}$.

Consider any edge (*u*,*v*) in the tree. To prove that (*u*,*v*) resolves a quartet not resolved by any other edge, we need to show that there exists a quartet *ab*|*cd* of the form shown in Figure 4. First, we describe how to select leaves *a* and *b*. Consider the following cases: 

1. *u* has at least two neighbors *i* and *j*, apart from *v*, that are internal nodes. Then, we select any $a\in M_{i}^{\text{ui}}$ and any $b\in M_{j}^{\text{uj}}$.

2. *u* has only one neighbor *i* ≠ *v* that is an internal node. Then, at least one of *u*’s neighboring leaves must participate in a quartet that (*u*,*v*) resolves. Without such a leaf, (*u*,*v*) would resolve the same set of quartets as (*u*,*i*), so one of these two edges would be contractible, contradicting the assumption that the tree is maximally reduced. We select this leaf as *b* and we select any $a\in M_{i}^{\text{ui}}$.

3. All neighbors of *u*, except *v*, are leaves. Then, at least two of its neighbors must participate in a quartet, because (*u*,*v*) must resolve a quartet. We select the two neighbors as *a* and *b*.

In every case, we can select the desired leaves *a* and *b*. By a similar argument, we can also select the desired *c* and *d*. This proves the existence of the desired quartet *ab*|*cd*. Therefore, each internal edge of *T* uniquely resolve a quartet. □

The next result shows that the set of quartets resolved by a maximally reduced tree uniquely identifies the tree.

### Theorem 3

*Let T and T*^′^*be two maximally reduced trees such that*$I(T)=I(T^{\prime })$. *Then, T and T*^′^*are isomorphic.*

The *maximally reduced form* (MRF) of a MUL-tree *T* is the tree that results from repeatedly pruning prunable leaves and contracting contractible edges from *T* until this is no longer possible. Theorem 3 shows that we can indeed talk about “the” MRF of *T*. Before proving Theorem 3, we mention some of its consequences.

### Corollary 1

Every MUL-tree has a unique MRF.

### Corollary 2

Any two MUL-trees with the same information content have the same MRF.

### Corollary 3

*If a maximally reduced MUL-treeTis not singly-labeled, there does not exist a singly-labeled treeT*^′^*such that*$I(T)=I(T^{\prime })$.

**Figure 5:**
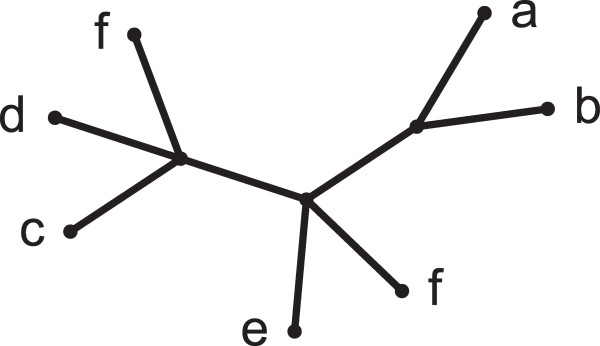
A maximally reduced MUL-tree.

Note that Corollary 3 does not contradict Theorem 1. If the MUL-tree in Theorem 1 is maximally reduced and not singly-labeled, the containment is proper; i.e., $I(T)\neq I(T^{\prime })$, which is the claim of Corollary 3. Figure 5 illustrates this. Any singly-labeled tree resolving the same set of quartets must be obtained by removing one of the leaves labeled with *f*. However, doing so will also introduce quartets that are not resolved by the maximally reduced MUL-tree.

### Corollary 4

The relation “sharing a common MRF” is an equivalence relation on the set of MUL-trees.

The last result implies that MUL-trees can be partitioned into equivalence classes, where each class consists of the set of all trees with the same information content. Thus, instead of comparing MUL-trees directly, we can compare their maximally reduced forms.

We now proceed to the proof of Theorem 3. We need two lemmas.

### Lemma 4

*There is a bijectionϕbetween the respective sets of internal edges of T and T*^′^*with the following property. Let* (*u*,*v*) *be an internal edge in T and let* (*u*^′^,*v*^′^) = *ϕ*(*u*,*v*). *Then*, $M_{u}^{\text{uv}}=M_{u^{\prime }}^{u^{\prime }v^{\prime }}$*and*$M_{v}^{\text{uv}}=M_{v^{\prime }}^{u^{\prime }v^{\prime }}$. *Therefore*, Δ(*u*,*v*) = Δ(*u*^′^,*v*^′^).

### Proof

**Figure 6:**
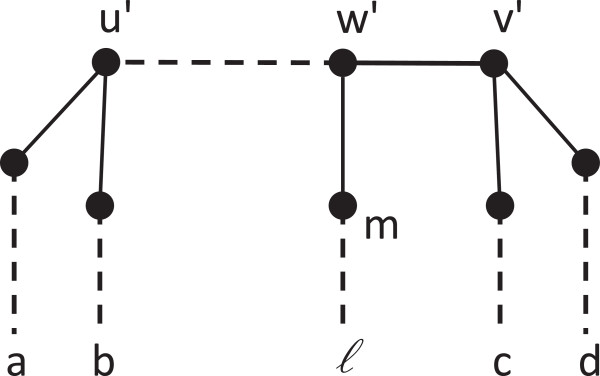
Supportive illustration for the proof of Lemma 4.

Consider an edge (*u*,*v*) in *T*. By Theorem 2, (*u*,*v*) must resolve a quartet *ab*|*cd* not resolved by any other edge as shown in Figure 4. We claim that this quartet must be resolved uniquely by an edge (*u*^′^,*v*^′^) in *T*^′^. Suppose not. Using arguments similar to those in the proof of Lemma 3, we can show that all edges that resolve *ab*|*cd* in *T*^′^ form a path (*u*^′^,*x*^′^,…,*w*^′^,*v*^′^), where possibly *x*^′^ = *w*^′^, as shown in Figure 6. Here, $\{a,b\}\subseteq M_{u^{\prime }}^{u^{\prime }x^{\prime }}$ and $\{c,d\}\subseteq M_{v^{\prime }}^{w^{\prime }v^{\prime }}$.

Since (*w*^′^,*v*^′^) resolves a quartet not resolved by any other edge, by Theorem 2 there exists a label *ℓ* as shown, where $\ell \in M_{m}^{w^{\prime }m}$. Since *ab*|*ℓd* is a quartet in *T*^′^ and I(T)=I(T), it must be true that $\ell \in M_{v}^{\text{uv}}$ in *T*. Clearly, *T* does not resolve the quartet on {*a*,*ℓ*,*d*,*c*} in the same way, *aℓ*|*cd*, as *T*^′^. This contradicts the assumption that $I(T)=I(T^{\prime })$. Thus, (*u*^′^,*v*^′^) must be an edge. Moreover, only one such edge exists in *T*^′^ as it uniquely resolves the quartet *ab*|*cd*.

Now consider any label $f\in M_{u}^{\text{uv}}$ such that *f* ∉ {*a*,*b*,*c*,*d*}. Label *f* must be in $M_{u^{\prime }}^{u^{\prime }v^{\prime }}$; otherwise, *T* and *T*^′^ would resolve the quartet {*a*,*f*,*c*,*d*} differently. Similarly, any such $f\in M_{u^{\prime }}^{u^{\prime }v^{\prime }}$ must be in $M_{u}^{\text{uv}}$ as well. Thus $M_{u}^{\text{uv}}=M_{u^{\prime }}^{u^{\prime }v^{\prime }}$. In the same way, we can prove that $M_{v}^{\text{uv}}=M_{v^{\prime }}^{u^{\prime }v^{\prime }}$. Thus, Δ(*u*,*v*) = Δ(*u*^′^,*v*^′^).

We have shown that there is a one-to-one mapping *ϕ* from edges in *T* to edges of *T*^′^ such that Δ(*e*) = Δ(*ϕ*(*e*)). To complete the proof, we show that *ϕ* is onto. Suppose that for some edge *e*^′^ in *T*^′^ there is no edge *e* in *T* such that *ϕ*(*e*) = *e*^′^. But then *e*^′^ must resolve a quartet not resolved by any other edge in *T*^′^. This quartet cannot be in I(T), contradicting the assumption that $I(T)=I(T^{\prime })$. □

Let *ϕ* be the bijection between the edge sets of *T* and *T*^′^ from the preceding lemma.

### Lemma 5

*Let* (*u*,*v*) *and* (*v*,*x*) *be any two neighboring internal edges inT*, *and let* (*p*,*q*) = *ϕ*(*u*,*v*) *and* (*r*,*s*) = *ϕ*(*v*,*x*) *be the corresponding edges in T*^′^*such that*$M_{u}^{\text{uv}}=M_{p}^{\text{pq}}$*and*$M_{v}^{\text{vx}}=M_{r}^{\text{rs}}$. *Then*, (*p*,*q*) *and* (*r*,*s*) *are neighbors in T*^′^*with q* = *r*.

### Proof

Since (*u*,*v*) and (*v*,*x*) are neighbors, and each resolves a quartet that is not resolved by the other, $M_{u}^{\text{uv}}\subset M_{v}^{\text{vx}}$ and $M_{v}^{\text{uv}}⊃M_{x}^{\text{vx}}$. By Lemma 4, this implies that $M_{p}^{\text{pq}}\subset M_{r}^{\text{rs}}$ and $M_{q}^{\text{pq}}⊃M_{s}^{\text{rs}}$. Thus, the only way (*p*,*q*) and (*r*,*s*) can exist in *T*^′^ is as part of the path $P_{p,s}=(p,q,\ldots ,r,s)$. If *q* ≠ *r*, then consider the edge (*t*,*r*) on *P*_*ps*_ such that *p* is closer to *t* than to *r*. Then, the following must hold:

(1) $$M_{p}^{\text{pq}}\subset M_{t}^{\text{tr}}\subset M_{r}^{\text{rs}}$$

and

(2) $$M_{q}^{\text{pq}}⊃M_{r}^{\text{tr}}⊃M_{s}^{\text{rs}}$$

Let (*z*,*w*) = *ϕ*^−1^(*t*,*r*) be the edge in *T* corresponding to (*t*,*r*). Irrespective of the position of (*z*,*w*) in *T*, (1) and (2) cannot be simultaneously true with respect to edges (*u*,*v*), (*v*,*x*) and (*z*,*w*) in *T*. Therefore, *q* = *r*, which proves the desired result. □

### Proof of Theorem 3

Lemmas 4 and 5 show that *T* and *T*^′^ are isomorphic with respect to their internal edges. It remains to show a one-to-one correspondence between their leaf sets. For this, we match up the leaves attached at every pendant node in *T* and *T*^′^. We start with pendant nodes to which only one internal edge is attached. For example, consider an internal edge (*u*,*v*) in *T* such that *v* is a pendant node and $T_{v}^{\text{uv}}$ has only leaves. Let (*u*^′^,*v*^′^) = *ϕ*(*u*,*v*) be the corresponding edge in *T*^′^ such that $M_{u}^{\text{uv}}=M_{u^{\prime }}^{\text{uv}}$. By Lemma 4, $C^{\text{uv}}=C^{u^{\prime }v^{\prime }}$. Moreover, neither *T* nor *T*^′^ have prunable leaves. Thus, the same set of leaves must be attached at *v* and *v*^′^ respectively. In subsequent steps, we select an internal edge (*u*,*v*) in *T* such that *v* is a pendant node and all the other pendant nodes in $T_{v}^{\text{uv}}$ have already been matched up in previous iterations. Again, let (*u*^′^,*v*^′^) = *ϕ*(*u*,*v*) such that $M_{u}^{\text{uv}}=M_{u^{\prime }}^{\text{uv}}$. Using similar arguments, the same set of leaves must be attached at *v* and *v*^′^ respectively. Proceeding this way, each pendant node in *T* can be paired with the corresponding pendant node in *T*^′^, and be shown to have the same set of leaves attached to them. This shows that *T* and *T*^′^ are isomorphic, as claimed. □

## Identifying contractible edges and prunable leaves

In preparation for the MUL-tree reduction algorithm of the next section, we give some results that help to identify contractible edges and prunable leaves.

The setting for the next result is the same as for Lemmas 2 and 3: (*u*,*v*) and (*w*,*x*) are two edges in tree *T* that lie on the path $P_{u,x}=(u,v,\ldots ,w,x)$ (see Figure 3). We say that subtree $T_{z}^{\text{yz}}$*branches out* from the path *P*_*u*,*x*_ if *y* ∈ *P*_*u*,*x*_−{*u*,*x*}, and *z* ∉ *P*_*u*,*x*_.

### Lemma 6

*Suppose* Δ(*u*,*v*) ⊆ Δ(*w*,*x*) *then*

1. *every internal edge on a subtree branching out from P*_*u*,*x*_*is contractible, and*

2. *if* Δ(*u*,*v*) = Δ(*w*,*x*), *every leaf on a subtree branching out from P*_*u*,*x*_*is prunable. Thus, the entire subtree can be deleted without changing the information content of the tree.*

### Proof

**Figure 7:**
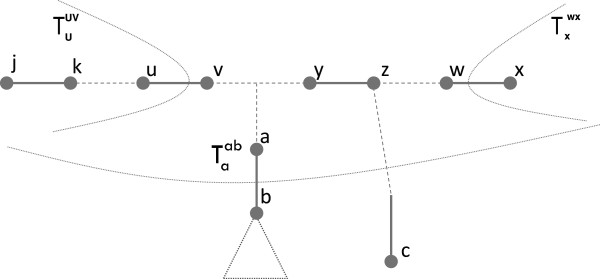
Supportive illustration for the proof of Lemma 6.

Refer to Figure 7. □

1. Consider any edge (*A*,*B*) in a subtree branching out of *P*_*u*,*x*_, as shown. We claim that $M_{a}^{\text{ab}}\cup C^{\text{ab}}=M$; i.e., all the labels in *M* appear in $T_{a}^{\text{ab}}$. This means that $M_{b}^{\text{ab}}=\emptyset$, so (*A*,*B*) is uninformative.

To prove the claim, observe first that, by definition, $M_{u}^{\text{uv}}\cup C^{\text{uv}}\cup M_{v}^{\text{uv}}=\mathrm{M.}$ By Lemma 2, since Δ(*u*,*v*) ⊆ Δ(*w*,*x*), we have $M_{x}^{\text{wx}}=M_{v}^{\text{uv}}$, so

(3) $$M_{u}^{\text{uv}}\cup C^{\text{uv}}\cup M_{x}^{\text{wx}}=M.$$

Now, $M_{u}^{\text{uv}}\cup C^{\text{uv}}$ is the set of labels on the leaves of $T_{u}^{\text{uv}}$, while every label in $M_{x}^{\text{wx}}$ appears in $T_{x}^{\text{wx}}$. Hence, $T_{u}^{\text{uv}}$ and $T_{x}^{\text{wx}}$ jointly contain every label in *M*. Since $T_{u}^{\text{uv}}$ and $T_{x}^{\text{wx}}$ are subtrees of $T_{a}^{\text{ab}}$, this completes the proof of the claim.

2. Suppose Δ(*u*,*v*) = Δ(*w*,*x*). By an argument similar to the one used in the proof of Lemma 3, we can show that any edge (*y*,*z*) on the path *P*_*v*,*w*_ = (*v*…*w*) (see Figure 7) satisfies $M_{v}^{\text{uv}}=M_{z}^{\text{yz}}=M_{x}^{\text{wx}}$ and $M_{u}^{\text{uv}}=M_{y}^{\text{yz}}=M_{w}^{\text{wx}}$. Consider a leaf *c* as shown; let *ℓ* be its label. Then, *ℓ* appears in $T_{x}^{\text{wx}}$, for else $M_{y}^{\text{yz}}\neq M_{w}^{\text{wx}}$, a contradiction. Similarly, *ℓ* appears in $T_{u}^{\text{uv}}$.

Now, let *S* be the tree obtained after pruning leaf *c*. 

(a) I(T)⊆I(S): Suppose pruning *c* removes a quartet from I(T). If such a quartet exists in *T*, it must be resolved by an edge $(j,k)\in T_{u}^{\text{uv}}$ (say). But then (*j*,*k*) still resolves the same quartet in *S* because $\ell \in M_{x}^{\text{wx}}$, and the labels in $T_{x}^{\text{wx}}$ are a subset of those in $T_{k}^{\text{jk}}$. This is a contradiction.

(b) I(S)⊆I(T): Suppose pruning *c* adds a quartet to I(S) that is not in I(T). Such a quartet in *S* must be resolved by an edge (*j*,*k*) in $S_{u}^{\text{uv}}$ (say), that before pruning satisfied *ℓ* ∈ *C*^*jk*^, but now has $\ell \notin M_{k}^{\text{jk}}$. However $\ell \in M_{x}^{\text{wx}}$; therefore we still have *ℓ* ∈ *C*^*jk*^ and the edge still cannot resolve the quartet, a contradiction.

Hence, *c* is prunable. □

### Lemma 7

*Suppose that T is a MUL-tree where no pendant node is adjacent to two or more leaves with the same label. Let ℓ be any multiply-occurring label in T and let T*^′^*be the minimal subtree of T that spans all the leaves labeled by ℓ. Then, any leaf in T labeled ℓ attached to a pendant node of degree at least three in T*^′^*is prunable.*

### Proof

**Figure 8:**
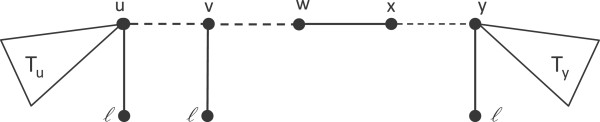
**Supportive illustration for the proof of Lemma 7.** The leaves attached to pendant nodes *u*, *v*, and *y* are labeled by *ℓ*, and the subtrees indicated by *T*_*u*_ and *T*_*y*_ do not contain a leaf labeled with *ℓ*. Nodes *u* and *y* have degree two in *T*^′^, while *v* has degree three.

Refer to Figure 8. Consider any pendant node *v* of degree at least three in *T*^′^ attached to a leaf labeled *ℓ*. Clearly deleting the leaf does not change the information content of any edge in *T*_*u*_ or *T*_*y*_. Now consider an edge (*w*,*x*) in *T*^′^ as shown. Note that *ℓ* ∈ *C*^*wx*^, so *ℓ* does not contribute to Δ(*w*,*x*). After deleting the leaf, we still have *ℓ* ∈ *C*^*wx*^, so Δ(*w*,*x*) remains unchanged. Therefore, the leaf is prunable. □

## The reduction algorithm

We now describe a *O*(*n*^2^) algorithm to compute the MRF of an *n*-leaf MUL-tree *T*. In the previous section, the MRF was defined as the tree obtained by applying information-preserving pruning and contraction operations to *T*, in any order, until it is no longer possible. For efficiency, however, the sequence in which these steps are performed is important. Our algorithm has three distinct phases: a preprocessing step, redundant edge contraction, and pruning of redundant leaves. We describe these next and then give an example.

### Preprocessing

For every edge (*u*,*v*) in *T*, we compute $\left|M_{u}^{\text{uv}}\right|$ and $\left|M_{v}^{\text{uv}}\right|$. This can be done in *O*(*n*^2^) time as follows. First, traverse subtrees $T_{u}^{\text{uv}}$ and $T_{v}^{\text{uv}}$ to count number of distinct labels $n_{u}^{\text{uv}}$ and $n_{v}^{\text{uv}}$ in each subtree. Then, $|M_{u}^{\text{uv}}|=|M|-n_{v}^{\text{uv}}$ and $|M_{v}^{\text{uv}}|=|M|-n_{u}^{\text{uv}}$. We then contract non-informative edges; i.e., edges (*u*,*v*) where $\left|M_{u}^{\text{uv}}\right|$ or $\left|M_{v}^{\text{uv}}\right|$ is at most one.

### Edge contraction and subtree pruning

Next, we repeatedly find pairs of adjacent edges (*u*,*v*) and (*v*,*w*) such that Δ(*u*,*v*) ⊆ Δ(*v*,*w*) or vice-versa, and contract the less informative of the two. By Lemmas 1 and 2, we can compare Δ(*u*,*v*) and Δ(*v*,*w*) in constant time using the precomputed values of $\left|M_{u}^{\text{uv}}\right|$ and $\left|M_{v}^{\text{uv}}\right|$. Lemma 6 (1) implies that we should also contract all internal edges incident on *v* or in the subtrees branching out of *v*. Further, by Lemma 6 (2), if Δ(*u*,*v*) = Δ(*v*,*w*), we can in fact delete these subtrees entirely, since their leaves are prunable. Lemma 3 implies that all such edges must lie on a path, and hence can be identified in linear time. The total time for all these operations is linear, since at worst we traverse every edge twice.

### Pruning redundant leaves

The tree that is left at this point has no contractible edges; however, it can still have prunable leaves. We first prune any leaf with a label *ℓ* that does not participate in any resolved quartet. Such an *ℓ* has the property that for every edge (*u*,*v*), $\ell \notin M_{u}^{\text{uv}}$ and $\ell \notin M_{v}^{\text{uv}}$. All such leaves can be found in *O*(*n*^2^) time and *O*(*n*) space.

Next, we consider sets of leaves with the same label *ℓ* that share a common neighboring pendant node. Such leaves can be found in linear time. For each such set, we delete all but one element. Let *T* be the tree that results from removing such leaves. Now, the only prunable leaves with a given label *ℓ* that might remain are leaves attached to different pendant nodes. By Lemma 7, we can identify and prune such leaves by performing the following steps. 

1. For each label *ℓ*, consider the subgraph on the leaves labeled by *ℓ*.

2. In this subgraph, delete any leaf not attached to a degree 2 pendant node as it is a redundant leaf.

This takes *O*(*n*) time per label and *O*(*n*^2^) time total. The space used is *O*(*n*). Hence, the overall time and space complexities are *O*(*n*^2^) time and *O*(*n*), respectively.

The resulting tree has no contractible edges nor prunable leaves. Therefore, it is the MRF of the orginal MUL-tree.

### An example

**Figure 9:**
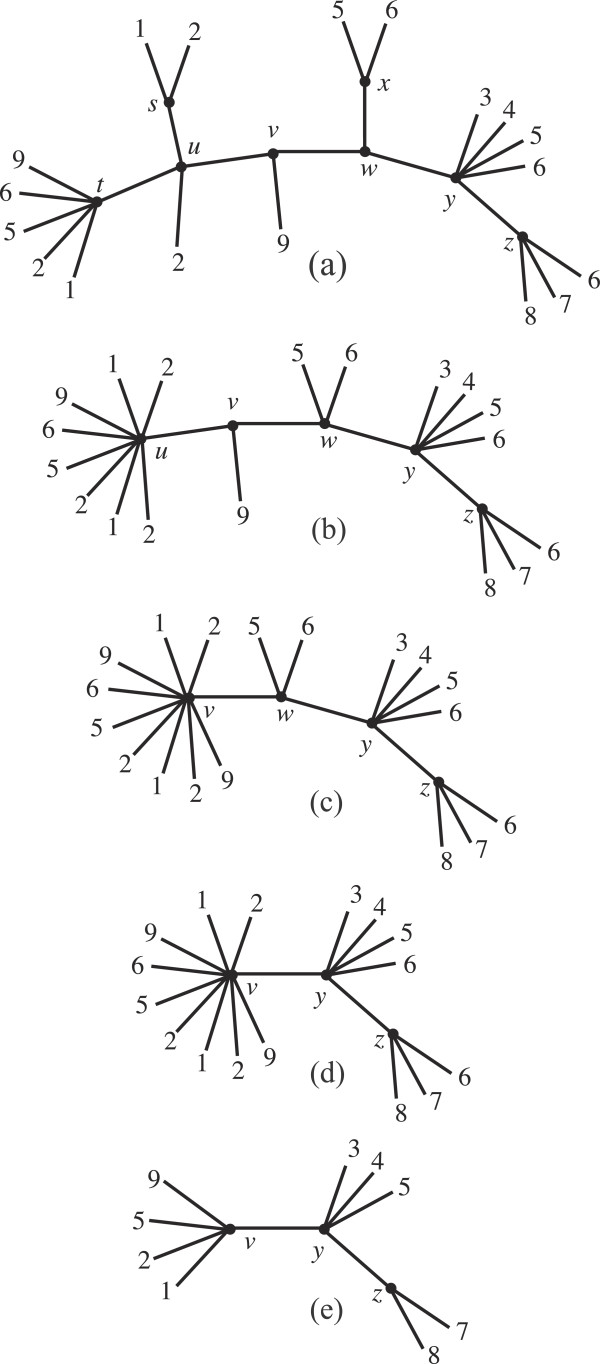
**Reduction to the MRF.** Supportive illustrations for the example on page 8.

We illustrate the reduction of the unrooted MUL-tree shown in Figure 9(a) to its MRF. 

1. In the preprocessing step, we find that $M_{t}^{\text{tu}}=\emptyset$, $M_{s}^{\text{su}}=\emptyset$ and $M_{x}^{\text{wx}}=\emptyset$, so edges (*t*,*u*), (*s*,*u*) and (*w*,*x*) are uninformative. They are therefore contracted, resulting in the tree shown in Figure 9(b).

2. Since Δ(*u*,*v*) ⊂ Δ(*v*,*w*), contract (*u*,*v*). The result is shown in Figure 9(c).

3. Since Δ(*v*,*w*) = Δ(*w*,*y*), delete the subtree branching out at *w* from the path from *v* to *y* and contract (*v*,*w*). The result is shown in Figure 9(d).

4. Prune taxon 6, which does not participate in any quartet, and all duplicate taxa at the pendant nodes. The result, shown in Figure 9(e), is the MRF of the original tree.

## Results and discussion

We implemented our MUL-tree reduction algorithm, as well as a second step that restricts the MRF to the set of labels that appear only once, which yields a singlylabeled tree. We tested our two-step program on a set of 110,842 MUL-trees obtained from the PhyLoTA database [6] ( http://phylota.net/; GenBank eukaryotic nucleotide sequences, release 184, June 2011), which included a broad range of label-set sizes, from 4 to 1500 taxa.

There were 8,741 trees (7.8%) with essentially no information content; these lost all resolution either when reduced to their MRFs, or in the second step. The remaining trees fell into two categories. Trees in set *A* had a singly-labeled MRF; 65,709 trees (59.3%) were of this kind. Trees in set *B* were reduced to singly-labeled trees in the second step; 36,392 trees (32.8%) were of this kind. Reducing a tree to its MRF (step 1), led to an average taxon loss of 0.83% of the taxa in the input MUL-tree. The total taxon loss after the second step (reducing the MRFs in set *B* to singly-labeled trees), averaged 12.81%. This taxon loss is not trivial, but it is far less than the 41.27% average loss from the alternative, naïve, approach in which all MUL-taxa (taxa that label more than one leaf) are removed at the outset. Note that, by the definition of MRFs, taxa removed in the first step do not contribute to the information content, since all non-conflicting quartets are preserved. On the other hand, taxa removed in the second step do alter the information content, because each such taxon participated in some non-conflicting quartet. Information content, in this case, will be lost but new information is never introduced, so the algorithm can be considered conservative.

**Figure 10:**
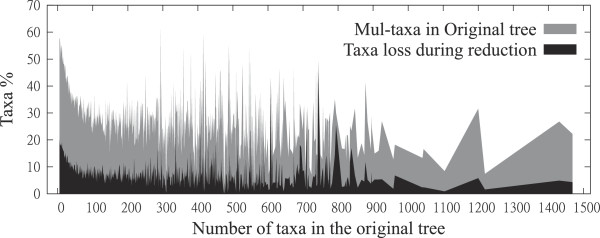
Experimental results: Taxon loss in the second step.

Taxon loss is sensitive to the number of total taxa and, especially, MUL-taxa, as demonstrated in Figure 10. The grey function shows the percentage of MUL-taxa in the original input trees, which is the taxon loss if we had restricted the input MUL-trees to the set of singly-labeled leaves. The black function shows the percentage of MUL-taxa lost after steps 1 and 2 of our reduction procedure.

**Figure 11:**
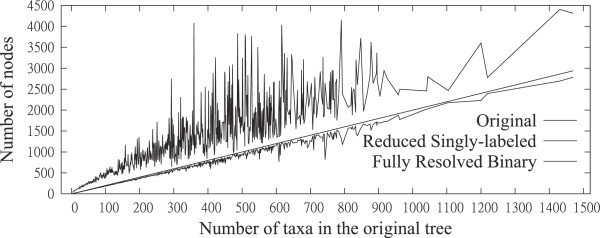
Experimental results: Quality of reduced singly-labeled trees.

In addition to the issue of taxon loss, we investigated the effect of our reduction on edge loss, i.e., the level of resolution within the resulting singly-labeled tree. Input MUL-trees were binary and therefore had more nodes than twice the number of taxa (Figure 11, solid line), whereas a binary tree on singly labeled taxa would have approximately as many nodes as twice the number of taxa (Figure 11, dashed line). We found that, although there was some edge loss, the number of nodes in the reduced singly-labeled trees (Figure 11, dotted line) corresponded well to the total possible, indicating low levels of edge loss. Note that each point on the dotted or solid lines represents an average over all trees with the same number of taxa.

We have integrated our reduction algorithm into STBase (available at http://stbase.org/), a phylogenetic tree search engine that takes a user-provided list of species names and finds matches with a precomputed collection of phylogenetic trees, more than half of which are MUL-trees, assembled from GenBank sequence data. The trees returned are ranked by a tree quality criterion that takes into account overlap with the query set, support values for the branches, and degree of resolution. We have added functionality to provide reduced singly-labeled trees as well as the MUL-trees based on the full leaf set and the label sets from the reduced singly-labeled trees are used in downstream supermatrix construction.

## Conclusions

We introduced an efficient algorithm to reduce a multi-labeled MUL-tree to a maximally reduced form with the same information content, defined as the set of non-conflicting quartets it resolves. We showed that the information content of a MUL-tree uniquely identifies the MUL-tree’s maximally reduced form. This has potential application in comparing MUL-trees by significantly reducing the number of comparisons as well as in extracting species-level information efficiently and conservatively from large sets of trees, irrespective of the underlying cause of multiple labels. Our algorithm can easily be adapted to work for rooted trees.

Further work investigating the relationship of the MRF to the original tree under various biological circumstances is also underway. We might expect, for example, that well-sampled nuclear gene families reduce to very small MRF trees, and that annotation errors in chloroplast gene sequences (in which we expect little gene duplication), result in relatively large MRF trees. Comparing the MRF to the original MUL-tree may well provide a method for efficiently assessing and segregating data sets with respect to the causes of multiple labels.

It would be interesting to compare our results with some of the other approaches for reducing MUL-trees to singly-labeled trees (e.g., [5]) or, indeed, to evaluate if our method can benefit from being used in conjunction with such approaches.

## Endnote

^a^The results presented here can be extended to rooted trees, using triplets instead of quartets, exploiting the well-known bijection between rooted and unrooted trees ( [23], p. 20).

## Competing interests

The authors declare that they have no competing interests.

## Authors’ contributions

MMM and DFB conceived the problem. AD, DFB and MMM designed the experiments and drafted the manuscript. AD designed and implemented the algorithms, and implemented the experiments. DFB coordinated the project. All authors read and approved the final manuscript.
